# Regulatory T Cells Kinetics in Immune Reconstitution Inflammatory Syndrome in HIV-Tuberculosis Co-Infected Individuals

**DOI:** 10.31083/JMCM25012

**Published:** 2025-02-05

**Authors:** Nicolas Barros, Cesar A. Sanchez, A. Clinton White, Allison Bauer, Fernando Woll, Edward A. Graviss, Carlos Seas, Eduardo Gotuzzo, Martin Montes

**Affiliations:** 1 Instituto de Medicina Tropical Alexander von Humboldt, Universidad Peruana Cayetano Heredia, 15102 Lima, Peru; 2 Division of Infectious Diseases, Indiana University, Indianapolis, IN 46202, USA; 3 Infectious Disease Division, Department of Internal Medicine, University of Texas Medical Branch, Galveston, TX 77555, USA; 4 Yale University, New Haven, CT 06511, USA; 5 Department of Pathology and Genomic Medicine, The Methodist Hospital Research Institute, Houston, TX 77030, USA

**Keywords:** regulatory T cells, IFN-*γ*, HIV, tuberculosis, immune reconstitution inflammatory syndrome, immune recovery

## Abstract

**Background:**

Combination antiretroviral therapy (cART) can suppress human immunodeficiency virus (HIV-1) replication, but some patients develop worsening of co-infections, termed immune reconstitution inflammatory syndrome. Regulatory T cells (Tregs) are a population of CD4^+^ T cells that modulate immune responses. We hypothesized that immune reconstitution inflammatory syndrome (IRIS) is associated with Tregs dysfunction.

**Methods:**

We prospectively enrolled antiretroviral naive HIV patients with co-infection with *Mycobacterium tuberculosis* (MTB; N = 26) or controls with no prior opportunistic infection (N = 10). We prospectively measured HIV viral load, CD4^+^ T cell count, regulatory T cell (CD4^high^, CD127^low-neg^, Foxp^3+^) proportion, and Interferon-*γ* (IFN-*γ*) response to MTB peptides before and after initiation of combination antiretroviral therapy.

**Results:**

Eleven of the MTB patients developed IRIS; 15 did not. IRIS patients had a lower proportion of Tregs at baseline compared to no-IRIS patients (HIV/no-OI and HIV/MTB no-IRIS), but the difference did not reach statistical significance (IRIS: 9.6 [5.3–11.2]; no-IRIS: 13.9 [7.6–22.5] *p* = 0.066). After 2 weeks of cART the proportion of Tregs was significantly lower in HIV/MTB IRIS patients (HIV/MTB IRIS: 9.8 [6.6–13.6], HIV/MTB no-IRIS: 15.8 [11.1–18.8]. The antigen-specific IFN-*γ* production was greater in the patients who developed IRIS compared with those who did not develop IRIS.

**Conclusion:**

IRIS patients had a lower proportion of Tregs and more marked IFN-*γ* production, suggesting that Tregs may be responsible for suppressing the antigen-specific inflammatory response.

## 1. Background

Combination antiretroviral therapy (cART) can suppress human immunodeficiency virus (HIV-1) replication, leading to decreased morbidity and mortality, but some patients develop unmasking of previously unrecognized or worsening of previously recognized co-infections. This has been termed immune reconstitution inflammatory syndrome (IRIS) [1–3]. IRIS is a common complication starting of cART, particularly in those who begin therapy with CD4 cell counts below 50 cells/mcL. The high prevalence of both HIV and *Mycobacterium tuberculosis* (MTB) in the developing world renders co-infected patients at a high risk for experiencing clinical deterioration [4,5].

Current data support initiating cART soon after MTB therapy in cases of HIV/MTB co-infection, but early initiation of cART increases the risk of IRIS [6–10]. The optimal strategy for management of IRIS has yet to be defined. Initially cART was interrupted, but the patients with advanced HIV disease were more susceptible to other infections [3]. Some authorities recommended delaying antiretroviral therapy, but recent studies demonstrate that delaying antiretroviral therapy may be associated with increased mortality [8,9,11]. Anti-inflammatory therapy has been used for IRIS, mild cases being treated with non-steroidal anti-inflammatory drugs and severe cases managed with corticosteroid therapy. Yet steroid treatment renders patients more susceptible to acquiring additional infections [12]. Furthermore, a number of IRIS cases remain diagnostically elusive. In MTB-HIV co-infection, IRIS can be confused with treatment failure, drug resistance, drug toxicity, or additional infections [13]. Understanding the immunopathogenesis of IRIS may provide alternative strategies for diagnosis and treatment [2].

Regulatory T cells (Tregs), a subpopulation of CD4^+^ lymphocytes, may be implicated in the pathogenesis of IRIS [14,15]. Tregs are a unique population of CD4^+^ T cells that are able to modulate immune responses to self-antigens and exuberant reactions to foreign antigens. The main group of Tregs, characterized by high constitutive expression of CD25 (Interleukin-2 (IL-2) receptor), modulate immune responses to self-antigens [16,17]. Tregs exert their suppressive function through the production of anti-inflammatory cytokines, cell-to-cell contact, and by consumption of pro-inflammatory cytokines. Tregs play an important role mediating peripheral tolerance and reducing deleterious immune responses [18,19]. HIV infects Tregs and it can affect both their number and function [20,21]. However, a recent study suggested that despite quantitative changes in Tregs, HIV-1 infection was not associated with an impairment of their *ex-vivo* suppressive function [22,23].

After initiation of cART, the kinetics of reconstitution of the various CD4^+^ subpopulations, including Tregs, may occur at differential rates. We and others have previously described that the proportion of regulatory T cells (CD4^+^Foxp3+ cells) are increased in cART naive patients and that this proportion seems to normalize over the course of cART [24–27]. However, in those with low CD4^+^ cell counts, we noted a wide range in the proportion of T cells expressing Treg markers from markedly increased proportions to slightly depressed levels. We hypothesized that the function and proportion of Tregs are reduced in patients who develop IRIS and this condition is associated with increased antigen-specific responses during the uncontrolled inflammatory syndrome.

## 2. Methods

### 2.1 Patients

This prospective study was performed at Instituto de Medicina Tropical “Alexander von Humboldt” in Lima, Peru, under a protocol approved by the Institutional Review Boards of Universidad Peruana Cayetano Heredia (Comité Institucional de Ética), the University of Texas Medical Branch. Healthy. HIV seronegative individuals (*n* = 16) were enrolled as controls for baseline CD4^+^ and Tregs. Patients who tested positive for HIV on two consecutive diagnostic tests (Enzyme-linked immunosorbent assay and Western Blot) were referred for cART and enrollment in the current study. Antiretroviral therapy naive patients who consented to participate in this study were enrolled from 2005 to 2009. Subjects included HIV-infected, cART naive at study entry, completing 48 weeks of clinical follow-up, who achieved viral suppression (*<*400 copies of viral RNA/mL). The HIV infected patients included a group with no active or previous opportunistic infection (HIV/no-OI). A second group had a diagnosis of MTB. Diagnosis of MTB was made by positive smear or culture (*n* = 17) in most patients. Nine patients initiated anti-MTB therapy based on clinical grounds and responded to therapy. Patients were further classified according to the development of IRIS (IRIS *vs.* no IRIS). Criteria for this diagnosis were gleaned from definitions utilized by other researchers studying IRIS [28]. Clinical features of IRIS diagnosis included: initiation of cART; virologic suppression of HIV during IRIS event; the emergence of an inflammatory response that could not be explained by the pathogenesis of existing infection; a new infection or side effects of treatment; or credit to a specific pathogen [28,29]. The classification of an inflammatory response after initiation of cART as IRIS was discussed and confirmed by the clinicians on the study team. Plasma samples were taken at baseline (pre-cART) and 2, 4, 8, 12, 24, 36, and 48 weeks after initiation of therapy to measure viral load and ensure virologic suppression over the course of treatment. Peripheral blood mononuclear cells were taken at baseline and at the same time points after initiating therapy to determine CD4 and Tregs proportions.

### 2.2 Flow Cytometry

Peripheral blood mononuclear cells (PBMCs) were isolated from heparinized blood within 6 hours by density gradient centrifugation (Ficoll-Hypaque GE Healthcare, Chicago, IL, USA). PBMCs were stained with surface markers using peridinin-chlorophyll-protein (PerCP) anti-CD4 and phycoerythrin-conjugated (PE) anti-CD25 and allophycocyanin (APC) CD127 monoclonal antibodies (BD Biosciences, La Jolla, CA, USA). After fixation and permeabilization, the cells were stained with intracellular Foxp3 using a fluorescein-isothiocyanate (FITC)-conjugated anti-Foxp3 monoclonal antibody (clone ECH 101, eBiosciences, San Diego, CA, USA). Cells were acquired using a FACScalibur flow cytometer (Beckton Dickinson, Franklin Lakes, NJ, USA). Regulatory T cells were identified as CD25^+high^, CD127^low-neg^ and expression of Foxp3+ among CD4^+^ cells within the lymphocyte gate. Absolute CD4^+^ cell counts were performed using a 4-color single-platform staining of whole blood cells (anti–CD3-FITC, CD4-PE, CD45 PerCP, and CD8 APC). Flow cytometry data were analyzed using Flowjo software version 8.5 (Becton, Dickinson and Company, Ashland, OR, USA). Data were analyzed using Prism GraphPad version 5 (GraphPad Software, La Jolla, CA, USA).

### 2.3 Measure of MTB-specific Responses

PBMCs were isolated from heparinized blood, washed with RPMI (Sigma, Research Triangle, NC, USA), and counted using trypan blue to determine viability. PBMCs were plated at a concentration of 1 × 10^6^ cells/mL in 96-well plates and incubated in complete medium (RPMI, 10% fetal bovine serum (Sigma, Research Triangle, NC, USA) and antibiotic supplement (Sigma, Research Triangle, NC, USA); 37 °C, 5% of CO_2_, 72 h) plus a pool of 4 M. tuberculosis peptides (ESAT-6 positions 31–50 and 61–80, CFP-10 positions 51–70 and 71–90 from Baylor Genomic and RNA Profiling Core, Houston, TX, USA). Supernatants were harvested and stored at −70 °C until assay by ELISA for Interferon-*γ* (IFN-*γ*) using a commercial kit (BD OptEIA Human IFN-*γ* ELISA Kit II, BD bioscience, San Diego, CA, USA). This interferon gamma release assay (IGRA) test was previously compared with a commercial IGRA test (QuantiFERON Gold TB). Similar results were obtained in both tests (data not published).

### 2.4 Statistical Analysis

A Fisher’s exact test was used to assess differences between patient groups. Kruskal-Wallis test was used to compare the number of CD4^+^ cells between HIV/no-OI patients, IRIS, and no-IRIS patients. We also used the Mann-Whitney test to assess the differences in the proportion of Tregs in the different time points. In order to discriminate between IFN-*γ* titers in the patients who developed IRIS and those who did not, the non-parametric statistical Mann-Whitney test was used. All data are presented as medians with interquartile ranges in brackets.

## 3. Results

### 3.1 Subjects

Sixty-four patients were enrolled in the study and nine were screened but not enrolled. Twenty-eight of those enrolled did not complete follow up (*n* = 23) or did not achieve viral suppression (*n* = 5). Thirty-six patients completed up to 48 weeks of clinical follow-up after cART initiation. Seventy-five percent of the subjects were male (27 male and 9 female). The median age at enrollment was 35 years old (interquartile range (IQR): 30–43). Among the HIV-infected patients, 10 had no previous or active opportunistic infection (HIV/no-OI) and 26 patients had MTB co-infection. Among co-infected patients, 11 met the established criteria for IRIS (HIV/MTB IRIS) and 15 did not develop IRIS during follow-up (HIV/MTB no-IRIS). None of the HIV/no-OI patients developed IRIS. Eighty-five percent of the cases of IRIS presented within the first 4 weeks after initiation of cART (median: 2 weeks [IQR: 1–3 weeks]). There were no significant differences between the HIV/MTB IRIS and HIV/MTB no-IRIS groups in the percentage of patients with microbiological confirmation of MTB infection (IRIS: 71%, non-IRIS: 63%, *p* = 0.34) or the timing from initiating MTB treatment and cART (IRIS: 1.5 months [0.75–3.0], non-IRIS: 3.75 months [2.25–5.25] *p* = 0.11).

### 3.2 T Cell Subset Proportions during cART

**Fig. 1.**
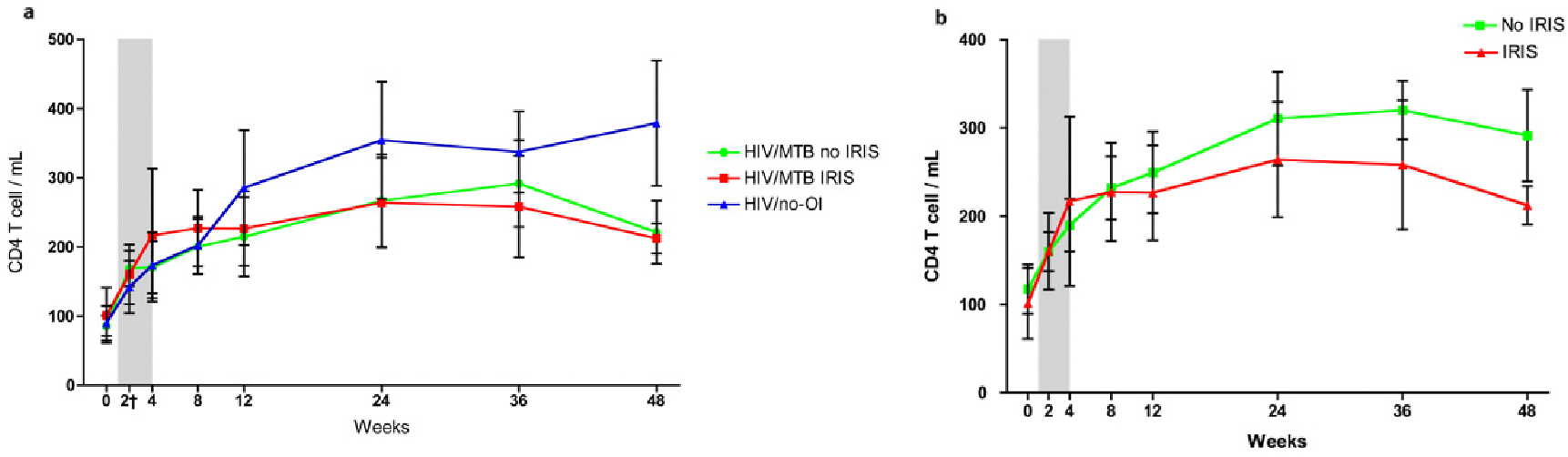
CD4^+^ T cell dynamics during cART. (a) During cART treatment all the patients increased their absolute numbers of circulating CD4^+^ T cells. No differences were found between HIV and MTB co-infected individual and HIV patients with no signs of opportunistic infections. (b) No differences were found when comparing the HIV patients that developed IRIS with those who did not. The gray area represents the time frame when most of the patients developed IRIS. The dots and lines represent means with the standard error of the mean in bars. HIV/MTB: HIV and MTB coinfected individuals that did not developed IRIS. HIV/MTB IRIS: HIV and MTB coinfected individuals that developed IRIS. HIV/no-OI: HIV infected patients with no signs or active or previous opportunistic infections. cART, Combination Antiretroviral Therapy; HIV, Human Immunodeficiency Virus; MTB, *Mycobacterium tuberculosis*; IRIS, Immune Reconstitution Inflammatory Syndrome; HIV/no-OI, HIV with no Opportunistic Infections.

**Fig. 2.**
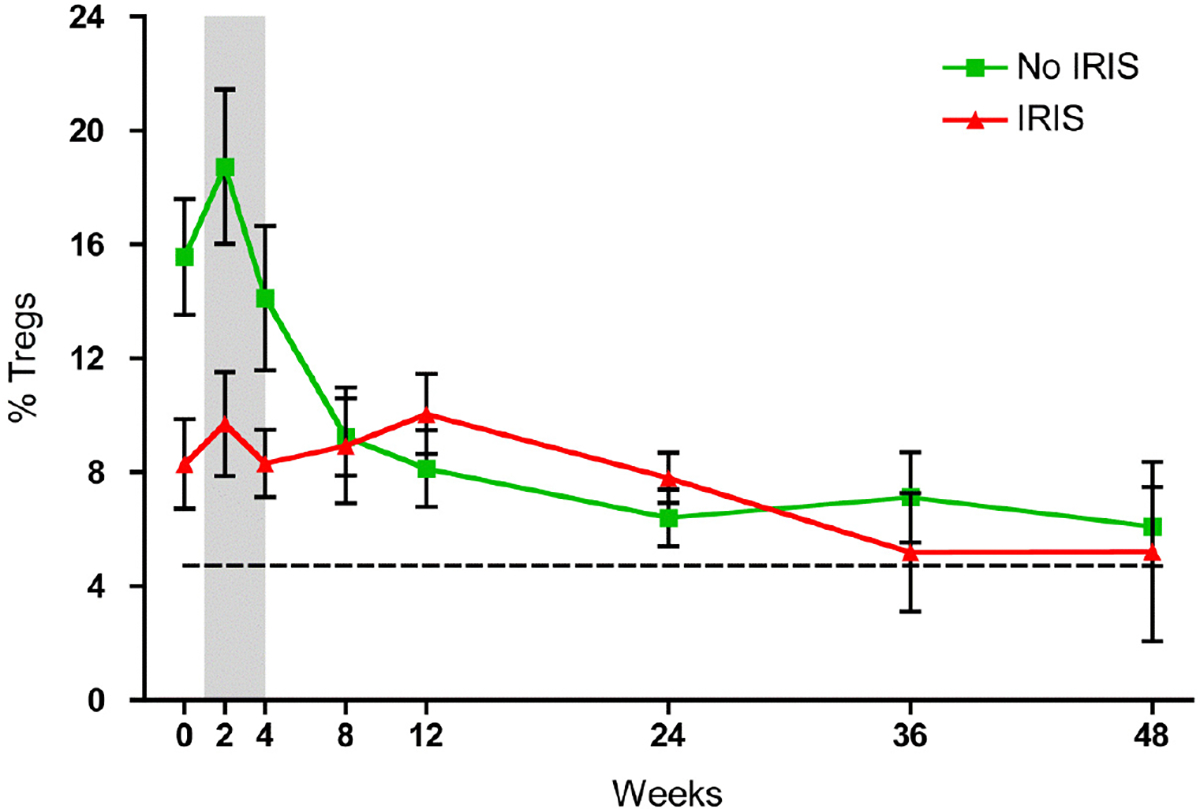
Regulatory T cell dynamics during cART. The proportion of circulating Tregs after 2 weeks of cART was diminished in the patients who developed IRIS when compared to those who did not (*p* = 0.0099). The gray area represents the time frame when most of the patients developed IRIS. The dashed line represents the median proportion of circulating Tregs in the healthy controls. The dots and lines represent means with the standard error of the mean in bars. no IRIS: HIV and MTB coinfected individuals that did not develop IRIS. IRIS: HIV and MTB coinfected individuals that developed IRIS.

Baseline CD4^+^ cell counts were similar for all groups at baseline (HIV/no-OI: 67 [range 27–166], HIV/MTB no-IRIS: 81 [range 47–115], HIV/MTB IRIS: 42 [range 28–116] *p* = 0.67). The increase in CD4^+^ cell counts during cART was similar in all the groups at all-time points (Fig. 1a,b). The percentage of CD4 cells expressing Tregs markers was increased in HIV infected patients from the three study groups compared to healthy individuals (HIV/MTB no-IRIS: 12.8 [6.8–21.8], HIV/MTB IRIS: 8.1 [1.2–11.6], HIV/no-OI: 15.6 [10.9–22.5], healthy seronegative individuals: 4.7 [3.4–5.7], *p* = 0.0002) [26]. IRIS patients had a lower proportion of Tregs at baseline compared to no-IRIS patients (HIV/no-OI + HIV/MTB no-IRIS), but the difference did not reach statistical significance (IRIS: 9.6 [5.3–11.2]; no-IRIS: 13.9 [7.6–22.5], *p* = 0.066). After 2 weeks of cART the proportion of Tregs was lower in HIV/MTB IRIS patients compared with HIV/MTB no-IRIS and HIV/no-OI patients (HIV/MTB IRIS: 9.8 [6.6–13.6], HIV/MTB no-IRIS: 15.8 [11.1–18.8], HIV/no-OI: 19.1 [11.4–21.5], *p* = 0.032). We found similar results when comparing all patients who did not developed IRIS (HIV/no-OI and HIV/MTB no-IRIS) with those who developed IRIS (IRIS: 9.8 [6.6–13.6], no-IRIS: 16.8 [11.3–21.2], *p* = 0.01). At 48 weeks after cART initiation, the proportion of Tregs slowly decreased and was similar with that of healthy seronegative individuals (HIV/MTB no-IRIS: 4.97 [3.4–5.2], HIV/MTB IRIS: 2.38 [1.78–11.5], HIV/no-OI: 7.1 [3.2–13.9], Healthy seronegative controls: 4.7 [2.5–5.7], *p* = 0.62) (Fig. 2).

Although there was a steady increase in the absolute numbers of circulating Tregs, no statistically significant differences were found between the study groups.

### 3.3 MTB Antigen-Specific Responses during cART

**Fig. 3.**
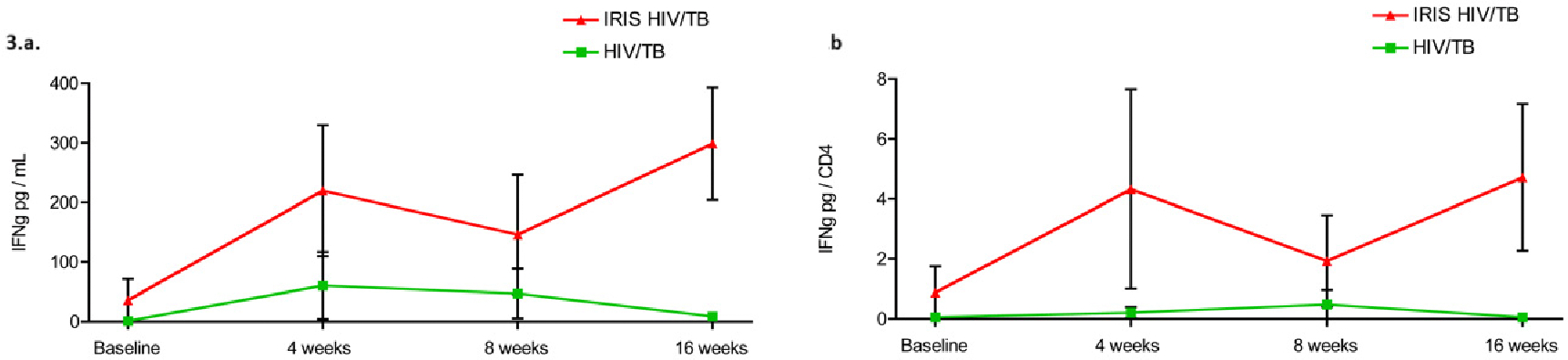
Antigen-specific Interferon-*γ* (IFN-*γ*) responses during cART. (a) HIV and *Mycobacterium tuberculosis* coinfected patients that developed IRIS presented with detectable antigen-specific IFN-*γ* responses throughout the treatment. In addition, they presented a larger expression of antigen specific IFN-*γ* responses between 8–16 weeks after cART when compared with the HIV and MTB coinfected individuals that did not developed IRIS (*p* = 0.01). (b) Similar results were found when comparing the expression of antigen specific IFN-*γ* responses per CD4^+^ T cells (*p* = 0.01). The dots and lines represent means with the standard error of the mean in bars. TB, tuberculosis.

MTB antigen-specific responses during cART were measured in the patients that were co-infected with MTB. The patients who developed IRIS demonstrated detectable antigen-specific IFN-*γ* production in all the time points. The antigen-specific IFN-*γ* production was greater in the patients who developed IRIS compared with those who did not develop IRIS and reached statistical significance at week 16 (Baseline, IRIS: median: 35.9 pg/mL (IQR 0.13–71.83), no-IRIS: 0 pg/mL [0–3.91]; week 4 IRIS: 323.3 pg/mL [0.04–336.6], no-IRIS: 5.93 pg/mL [0.41–229.5], week 8 IRIS: 1.75 pg/mL [0.75–332], no-IRIS: 1.12 pg/mL [0.52–140.3]; week 16: IRIS: 272 pg/mL [46.4–535.4], no-IRIS: 3.9 pg/mL [0–7.7], *p* = 0.02) (Fig. 3a,b).

## 4. Discussion

In this prospective, longitudinal study, we examined the reconstitution of the immune system’s regulatory T cells (Tregs) during cART in HIV+ patients coinfected with *Mycobacterium tuberculosis* (MTB). At baseline, the proportion of Tregs was increased in all HIV infected populations as has been previously shown [25–27,30]. There are conflicting data on the role of Tregs in the pathogenesis of HIV and IRIS [31]. While Tregs may influence the level of immune activation, they may suppress HIV-1–specific immune responses [32,33]. It is unclear if the increased proportions of Tregs found in HIV individuals may be a result of regulatory response to ongoing chronic inflammation. Over the first year on cART, the proportion of Tregs decreased until it reached similar levels to those of the healthy controls.

These findings confirm the fact that effective cART reconstitutes immune cells depleted during HIV infection to near normal levels [30,34,35]. For IRIS patients, the proportion of Tregs was significantly decreased 2 weeks after starting cART, suggesting that the balance of reconstitution between inflammatory and regulatory subsets may be unequal during the early phases of cART, which may drive an exuberant inflammatory response.

The higher production of IFN-*γ* in IRIS patients when compared to no-IRIS controls throughout treatment (Fig. 3a,b) implies that IRIS patients have more pronounced inflammatory responses, as has been previously demonstrated by other groups [14,28,36,37].

Our data differ somewhat from that of other groups that have studied Tregs in IRIS. Antonelli and colleagues did not find statistically significant differences in the proportion of CD4^+^ T cell subpopulations before or after reconstitution [28]. Though the baseline number of Tregs was lower in the IRIS group, the results were not statistically significant. Both Antonelli and colleagues and Tan and colleagues studied cryopreserved cells [28,37]. We and others have demonstrated that the expression of markers of Tregs (e.g., CD4^+^CD25^+^CD127^low^ and CD4^+^CD25^+^FoxP3^+^) is variably altered by cryopreservation [38,39]. Because of this variability, studies which used cryopreserved cells were likely underpowered to detect subtle differences.

Zaidi and colleagues [36] studied fresh cells. They also noted a lower proportion of Tregs at baseline in IRIS patients, but the difference was not statistically significant. However, when expressed as a total number of cells, the value for total number of Tregs was significantly lower.

Meintjes and colleagues [40] studied Tregs in HIV-MTB coinfected patients at the time of IRIS. They did not detect differences in expression of Foxp3 between those with IRIS and those without IRIS. Their definition of Tregs (CD4^+^Foxp3^+^), however, may also have included activated effector cells (CD4^+^CD25^−^Foxp3^+^).

None of the other groups collected data at both baseline and the 2-week time point. Since most IRIS events took place within the first four weeks of initiation of cART, changes in reconstituted immune populations that occurred in the first weeks after initiation of cART may have gone undetected.

Interestingly, despite the abnormal proportion of Tregs among HIV+IRIS patients during the first 4 weeks, the proportion slowly returned to normal levels (5%) in all HIV infected patients.

Our findings suggest that the balance between effector and regulatory immune mechanisms likely plays a key role in IRIS, and that IRIS may be due in part to insufficient immune regulation to blunt exuberant inflammatory responses. An important limitation of our study is the relatively small sample size which makes the generalization of the results limited. Further studies with a large population are needed to confirm our findings. In addition, further studies are needed to define the functional role of regulatory T cells in controlling activated responses to opportunistic infections.

## 5. Conclusion

Patients with HIV and MTB coinfection who developed IRIS had a lower proportion of Tregs and more marked MTB-specific IFN-*γ* production, suggesting that the balance between effector and regulatory immune mechanisms likely plays a key role in IRIS, and that IRIS may be due in part to insufficient immune regulation to blunt exuberant inflammatory responses.

## Data Availability

Any raw data and material discussions are available upon reasonable request to the corresponding author.
